# Glycerol Enhances the Antifungal Activity of Dairy Propionibacteria

**DOI:** 10.1155/2010/430873

**Published:** 2011-01-20

**Authors:** Helena Lind, Anders Broberg, Karin Jacobsson, Hans Jonsson, Johan Schnürer

**Affiliations:** ^1^Department of Microbiology, Swedish University of Agricultural Sciences, P.O. Box 7025, 750 07 Uppsala, Sweden; ^2^Department of Chemistry, Swedish University of Agricultural Sciences, P.O. Box 7015, 750 07 Uppsala, Sweden

## Abstract

Dairy propionibacteria are widely used in starter cultures for Swiss type cheese. These bacteria can ferment glucose, lactic acid, and glycerol into propionic acid, acetic acid, and carbon dioxide. This research examined the antifungal effect of dairy propionibacteria when glycerol was used as carbon source for bacterial growth. Five type strains of propionibacteria were tested against the yeast *Rhodotorula mucilaginosa* and the molds *Penicillium commune* and *Penicillium roqueforti*. The conversion of ^13^C glycerol by *Propionibacterium jensenii* was followed with nuclear magnetic resonance. In a dual culture assay, the degree of inhibition of the molds was strongly enhanced by an increase in glycerol concentrations, while the yeast was less affected. In broth cultures, decreased pH in glycerol medium was probably responsible for the complete inhibition of the indicator fungi. NMR spectra of the glycerol conversion confirmed that propionic acid was the dominant metabolite. Based on the results obtained, the increased antifungal effect seen by glycerol addition to cultures of propionibacteria is due to the production of propionic acid and pH reduction of the medium.

## 1. Introduction

During storage of grains, fruits, vegetables, silage, and processed foods, the contamination with molds and yeasts may cause spoilage, which can be associated with substantial economic losses and potential health hazards. To extend the shelf life of susceptible food and feed, considerable amounts of chemical preservatives are used. The development of natural preservatives, consisting of microorganisms generally recognized as safe (GRAS), might form an alternative to the chemicals. Both lactic acid bacteria (LAB) and propionibacteria are safe, well-characterized bacteria, commonly used in many industrial processes [1, 2]. The antifungal activity of LAB (reviewed in [3]) is explored in both applied systems [4–6] and at substance level [7–9]. The use of propionibacteria as a biopreservative culture has been tested alone [10, 11] and also in combination with LAB [12–15]. Propionibacteria are also found to produce antimicrobial compounds [16–18]. 

Glycerol is a colorless, odorless liquid, widely used in a great number of applications (e.g., skin care products and drug solvents). The rapid increase in biodiesel production seen in recent years [19] provides an abundant and inexpensive source of glycerol as a residue.

Previously, studies on the antifungal activity of LAB found that addition of glycerol enhanced the antifungal effect of certain species [20]. In LAB, the coenzyme B_12_-dependent glycerol/diol dehydratase is involved in the conversion of glycerol into 1,3-propanediol. An intermediate in the process is 3-hydroxypropionialdehyde, also known as reuterin, a potent antimicrobial compound. The presence of a diol dehydratase in *Propionibacterium freudenreichii* has been demonstrated [21], but a comparison with a number of species belonging to the genera *Enterobacteriaceae* indicated that the diol dehydratase of *P. freudenreichii* is different from the enterobacteria enzyme [22]. Growth of propionibacteria on glycerol increases propionic acid production [23], but the effect on the antifungal activity of these bacteria when using glycerol as single carbon source has not been previously described.

This study aimed at investigating the effect of glycerol on the antifungal activity of five different dairy propionibacteria species, as well as their metabolite profile when grown in the presence of glycerol. NMR spectroscopy was used to elucidate the conversion pathway and the end products, using ^13^C-labeled glycerol fed to cultures of propionibacteria.

## 2. Materials and Methods

### 2.1. Microorganisms, Media, and Growth Conditions

The propionibacteria used in this study were the type strains of the following dairy propionibacteria species, obtained from Deutsche Sammlung von Mikroorganismen und Zellkulturen GmbH (DSMZ): *Propionibacterium acidipropionici* DSMZ 4900, *P. freudenreichii* subsp. *shermanii* DSMZ 4902, *P. freudenreichii* subsp*. freudenreichii* DSMZ 20271, *P. thoenii* DSMZ 20276, and *P. jensenii* DSMZ 20535. The fungi used in the overlay assay and the microtitre plate assay were the yeast *Rhodotorula mucilaginosa* J350 (CFSQE 63) and the molds *Penicillium commune* J236 (IBT 10763) and *Penicillium roqueforti* J268 (IBT 6754). All fungi are part of the culture collection of the Department of Microbiology, Swedish University of Agricultural Sciences.

Cultures of propionibacteria were grown on modified sodium lactate (SL) medium (1% sodium lactate; Sigma-Aldrich, Steinheim, Germany, 1% tryptone; Oxoid Ltd., Hampshire, England, 0.5% yeast extract; Oxoid Ltd. and 0.5% KH_2_PO_4_) at 30°C in anaerobic jars (BBL, Becton, Dickinson and Co., Sparks, MD, USA) under CO_2_ + N_2_ atmosphere (GasPak System, BBL, Becton, Dickinson and Co). For growth experiments with glycerol, the same medium was used, but the sodium lactate was replaced by 1% glycerol (Merck, Darmstadt, Germany) (GLY). The yeast and molds were kept on malt extract (ME) agar (Oxoid Ltd.) slants at 2°C. Before use, the yeast was cultured in ME broth (Becton, Dickinson and Co.) on a rotary shaker (Infors AG, Bottmingen-Basel, Switzerland) at 120 rpm at 25°C overnight, while the molds were surface spread on fresh ME agar (Oxoid Ltd.) slants and incubated at 25°C until sporulation (3-4 days). The conidia (asexual spores) were then harvested from the slants using sterile peptone water. Yeast cells and mold conidia were counted using a Bürker counting chamber (Marienfeld, Lauda-Koenigshofen, Germany).

### 2.2. Antifungal Overlay Assay

The propionibacteria were inoculated in two parallel 2-cm streaks on SL plates (six per strain) and incubated at 30°C under anaerobic conditions for 72 hours. Soft agar (0.15% malt extract (Becton, Dickinson and Co.) and 1% agar base (Oxoid Ltd.)) with different concentrations of glycerol (0, 10, 50, 100, 200, and 500 mM) was cooled to 45°C before fungal cells or conidia were added to obtain a concentration of 10^4^ spores*ml^−1^. The soft agar was then poured onto the plates to overlay the SL agar. After incubation at 30°C for 48 to 72 hours under aerobic conditions, the plates were examined visually.

### 2.3. Acid Extraction from “Overlay” Plates

The plates were prepared in almost the same way as for the overlay assay, but the overlaid soft agar contained either 0 mM or 100 mM glycerol and no fungal cells or conidia were added. After the second incubation (48 hours at 30°C under aerobic conditions), the agar between the bacterial streaks was removed with a scalpel and mixed with sterile water to achieve a dilution of 1 : 10. The fluid was homogenized in a laboratory blender (Stomacher 400, Seward Ltd., Worthington, UK) for 60 seconds, followed by a soaking period of 30–60 minutes. The fluid was transferred to vials and centrifuged (4500 rpm, 10 minutes) to obtain agar-free liquid. Supernatants were filtered through a 0.45 *μ*m filter (Nalgene Nunc Int., Rochester, NY, USA) and analyzed by high-performance liquid chromatography. Standard solutions of lactic, acetic, and propionic acids at concentrations of 0.1, 0.5, 1.0, 5.0, 10, and 15 mM were included in the analysis, performed on a C-18 column (Zorbax SB-C18, Agilent Technologies, Waldbronn, Germany) at 30°C. As mobile phase 20 mM H_3_PO_4_, at a flow rate of 1.0 ml min^−1^, was used and the eluate was monitored with a UV detector (Agilent 1100 series, Agilent Technologies) at 210 nm. All experiments were repeated four times, and the values presented are the mean values.

### 2.4. Liquid Cultures

Propionibacteria were counted using a Petroff-Hausser cell counter (Hausser Scientific Partnership, Horsham, PA) and inoculated to achieve a final concentration of 10^7^ cells*ml^−1^ in SL and GLY broth (four replicates for each combination). The cultures were incubated as still cultures at 30°C for 72 hours. For viable bacterial count, one ml aliquots were serial diluted (1 : 10) six times with sterile peptone water, and 100 *μ*l of the two lowest dilutions were spread on SL agar plates and incubated at 30°C under anaerobic conditions. The remainings of the fermented broths were centrifuged (4500 rpm, 10 minutes) and filtered through 0.45 *μ*m filter (Nalgene Nunc Int.) to obtain cell-free supernatant. After tenfold dilution, the samples were analyzed by HPLC as described above. All experiments were repeated four times, and the values presented are the mean values.

### 2.5. Antifungal Screening in Microtitre Plates

The supernatants from the liquid cultures were also tested for antifungal activity using a microtitre plate assay. For each culture, two wells were prepared by adding 50 *μ*l of supernatant to 50 *μ*l of a cell or conidium suspension (prepared as previously described) to achieve a final concentration of 5 × 10^4^ ml^−1^. Uninoculated SL, GLY, and ME media were used as controls (50 *μ*l of broth + 50 *μ*l of cell or conidium suspension). The plates were incubated at 30°C in plastic bags, supplemented with a moist paper tissue to maintain humidity, and visually examined for fungal growth inhibition after 48 to 72 hours.

### 2.6. Correlation between Growth Stage and Antifungal Activity

To correlate the growth stage and antifungal activity, two representative propionibacteria strains were selected. Fresh cultures of *P. freudenreichii *subsp. *freudenreichii* and *P. jensenii *were washed in 0.8% NaCl solution and counted using a Petroff -Hausser counting chamber. A concentration of 10^7^ bacteria ml^−1^ was inoculated in GLY broth and incubated anaerobically as still cultures at 30°C. After 2, 8, 12, 24, 36, 48, 56, 72, 80, and 98 hours, 1 ml samples were withdrawn, and one aliquota was diluted and counted using a Petroff -Hausser counting chamber. The remaining of the sample was centrifuged (12 000 rpm, 10 min) and the supernatant was collected and kept at –20°C until further analysis. The supernatants were then diluted with sterile water two, four, and eight times. Aliquots (50 *μ*l) of all dilutions were added to two microtitre plate wells containing 50 *μ*l of a *P. roqueforti *conidial suspension. Eight wells were also prepared with 50 *μ*l ME broth and 50 *μ*l conidial suspension as growth controls. The final supernatant concentration in the wells was thus 50% (for the pure supernatant), 25%, 12.5%, and 6.25% and the conidium concentration was 5 × 10^4^ ml^−1^. Plates were incubated at 30°C in plastic bags containing a moist paper tissue to maintain humidity. After 48 hours, the plates were examined visually, and the degree of inhibition was compared to control wells and rated according to the following scale: 0, no inhibition, 1, weak visible inhibition, 2, obvious inhibition, 3, strong inhibition, but still visible growth, and 4, complete inhibition. The experiment was repeated once.

### 2.7. Fermentation with ^13^C-Labeled Glycerol

The glycerol conversion pathways in propionibacteria were studied by growing the cells in ^13^C-labeled glycerol, and analyzing the spent growth medium with NMR. Based on results from the previous experiment, *P. jensenii* was selected for this evaluation. The setup was the same as in the correlation experiment above, except for the medium which contained ^13^C-labeled glycerol (50% 1,3-labeled and 50% 2-labeled; Larodan Fine Chemicals AB, Malmö, Sweden) instead of glycerol. Samples were collected at 0, 24, 48, and 96 hours. The cell number was counted, and the remaining of the sample was centrifuged (12 000 rpm, 10 min), filtered (0.45 *μ*m), and frozen at −20°C until further analysis. The supernatants were analyzed for organic acids and low-molecular-mass metabolites with HPLC and ^13^C NMR spectroscopy. The HPLC analysis was performed on a cation exclusion column (Rezex ROA-Organic Acid, Phenomenex Inc., Torrance, CA, USA) at 25°C, using 5 mM H_2_SO_4_ as mobile phase at a flow rate of 0.6 ml min^−1^. The eluate was monitored with a refractive index detector (Agilent 1100 series, Agilent Technologies). Standard solutions of lactic, acetic, and propionic acid at concentrations of 10, 25, 50, and 100 mM were included in the analysis. The ^13^C NMR samples were prepared by mixing aliquots (600 *μ*l) of each supernatant with D_2_O (100 *μ*l). The samples were analysed by ^13^C NMR spectroscopy (100 MHz) at 30°C on a Bruker DRX-400 NMR spectrometer (Bruker Biospin GmBH, Rheinstetten, Germany) equipped with a 5-mm QNP probe head. Data from 128 scans were accumulated, and the spectral width was 240 ppm. The ^13^C NMR data were referenced against the glycerol CH_2_OH signal at *δ*_C_ 66.9. The pH of the supernatants was recorded using a PHM92 pH meter (Radiometer, Copenhagen, Denmark).

### 2.8. Statistical Analysis

Differences in bacterial growth (when monitored), pH, and acid production were analyzed for each propionibacteria when grown in different media, for both agar and broth methods, by two-way analysis of variance using Bonferroni posttests (Prism4, Graph Pad software). The level of significance was set to 5%.

## 3. Results

### 3.1. Antifungal Overlay Assay

**Figure 1:**
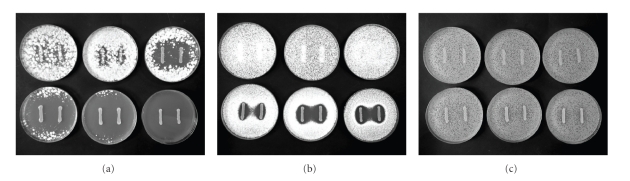
Antifungal overlay assay with *P. freudenreichii *subsp. *freudenreichii *against the molds *P. commune *(a),* P. roqueforti *(b), and the yeast *R. mucilaginosa *(c). Concentrations of glycerol in the overlaid agar are 0, 10, and 50 mM in the upper row from left to right and 100, 200, and 500 mM in the lower row from left to right for each picture.

All five type strains of propionibacteria were tested against three different target fungi (two molds and one yeast) at glycerol concentrations between 0 mM and 500 mM. The growth of both molds tested was affected by *P. freudenreichii* subsp. *freudenreichii *(Figure 1), as well as by the other tested propionibacteria (results not shown) when the bacteria was grown in the presence of glycerol, with increasing concentrations of glycerol enhancing the antifungal effect. The indicator mold *Penicillium commune *was the most sensitive fungi tested, and it was completely inhibited in the presence of 500 mM glycerol by most propionibacteria strains. *Penicillium roqueforti* was only slightly inhibited by *P. acidipropionici* and *P. thoenii* even at high glycerol concentrations, but with *P.* *freudenreichii* subsp. *freudenreichii *(Figure 1), *P. freudenreichii* subsp. *Shermanii, *and *P. jensenii *noticeably increased their inhibition with the addition of glycerol. The yeast *R. mucilaginosa* was the most resistant organism to the inhibitory activity of propionibacteria. From the results obtained, only *P. jensenii* showed visible inhibitory effects against *R. mucilaginosa* in the presence of glycerol, at concentrations of 200 and 500 mM (results not shown).

### 3.2. End-Product Analysis in Overlay Plate Experiments

The results of the acid analysis and pH measurements from the agar extractions are shown in Table 1. To evaluate the extraction efficiency of propionic acid from the agar, the analysis was first performed with plates containing 50 and 100 mM pure propionic acid. The procedure proved itself to be adequate since similar amounts of propionic acid were quantified in the extracts analyzed (data not shown). From plates without bacteria, only lactic acid (120 mM) and/or glycerol (30 mM) were extracted from the medium. The initial pH of the uncultured plates, with or without glycerol, was 5.5. The comparison between plates with and without glycerol for each propionibacterium revealed significant difference in the remaining amount of lactic acid only for *P. jensenii *(*P* < .01) with a smaller amount remaining on the plates without addition of glycerol. *P. thoenii* also showed a tendency towards less lactic acid remaining on plates without glycerol, but since the results from glycerol overlaid plates represented only two measurements, no reliable statistical analysis could be done. For *P. freudenreichii* subsp. *freudenreichii* and *P. freudenreichii* subsp. *Shermanii, *there was a significant difference in production of propionic acid, with higher amounts produced in the presence of glycerol (*P* < .001 and *P* < .05, resp.). The content of glycerol decreased in the medium after bacterial growth for all strains, but to a minor extent with *P. acidipropionici*. There was a significant difference in the pH for all of the strains when their growth in the presence of glycerol was compared (*P* < .001).

### 3.3. End-Product Analysis of Propionibacteria Grown in Liquid Medium

Production of acids and change in pH were recorded for the five propionibacteria strains grown in SL and GLY broths (Table 2). The initial pH of uncultured SL broth was 5.5, while GLY broth had a pH of 6.0. Lactic and acetic acid were only detected in cultures of SL medium and the production of propionic acid was significantly higher in these cultures (*P* < .001 for *P. freudenreichii* subsp. *shermanii*,* P. freudenreichii* subsp. *Freudenreichii,* and *P. jensenii* and *P* < .01 for *P. acidipropionici*) with the exception of *P. thoenii *(*P* > .05). Final counts cfu ml^−1^ did only differ significantly between SL and GLY for *P. freudenreichii* subsp. *freudenreichii* (*P* < .001). The final pH was noticeably lower in the GLY medium (*P* < .001 for all strains) (Table 2).

### 3.4. Antifungal Screening in Microtitre Plates

The supernatants from the liquid culture experiment were tested for antifungal activity in a microtitre plate assay (Table 2). Supernatants from cultures grown in SL medium, did not inhibit growth of any of the tested fungi, while growth of propionibacteria in GLY medium gave a clear inhibitory effect. *R. mucilaginosa *was completely inhibited by the supernatants of GLY cultures from all propionibacteria. The molds were inhibited by *P. acidipropionici, P. thoenii,* and *P. jensenii*, but not by *P. freudenreichii* subsp. *freudenreichii* or *P. freudenreichii* subsp. *shermanii, *even when GLY broth was used for bacterial growth. All three fungi were able to grow in uncultured SL and GLY broths (data not shown).

### 3.5. Correlation between Growth Stage and Antifungal Activity

**Figure 2:**
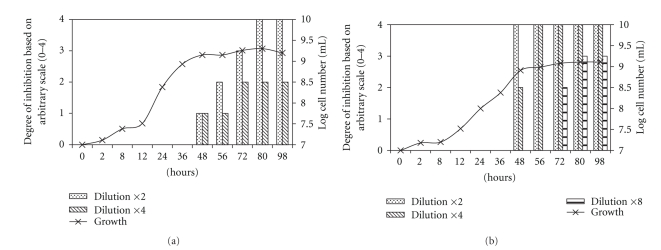
Growth curves and inhibition scores against *P. roqueforti* for the cell-free supernatant of (a) *P. freudenreichii *subsp. *freudenreichii* and (b) *P. jensenii*. The degree of inhibition showed in the graphs was determined by comparison to control wells and assigned according to the following scale: 0, no inhibition, 1, weak, visible inhibition, 2, obvious inhibition, 3, strong inhibition, but still visible growth and 4, complete inhibition.

The antifungal activity of diluted culture filtrates from *P. freudenreichii* subsp. *freudenreichii* and *P. jensenii* against *Penicillium roqueforti *was evaluated during bacterial growth, and the results can be seen in Figure 2. No antifungal activity was detected before the stationary growth phase (48 h) was achieved. However, after 48 h of incubation, the effect increased with time, reaching the highest level at 80 h. To demonstrate the increasing antifungal activity of the supernatants with time, they were diluted in sterile water two, four, and eight times. The antifungal activity of culture filtrates from *P. freudenreichii* subsp. *freudenreichii* showed “weak inhibition” (1 on the scale) against *Penicillium roqueforti* after 48 h of incubation when twofold diluted. At this dilution, maximum inhibition was seen at 80 h. When the culture filtrate was diluted four times, inhibition of *Penicillium roqueforti* was weak even at 56 h but increased after 72 h and was maintained at the higher level throughout the experiment. *P. jensenii *showed stronger antifungal activity earlier than *P. freudenreichii* subsp. *freudenreichii*. When the culture filtrate of *P. jensenii *was diluted two times, “complete inhibition” (4 on the scale) was seen already at 48 h. With a fourfold dilution of the same culture filtrate, complete inhibition was reached at 56 h. When it was diluted eight times, clear inhibition of *Penicillium roqueforti *was recorded after 72 h, with maximum inhibition achieved at 80 h and maintained until the end of the experiment. No further increase in inhibitory activity was observed between 80 and 98 h. The experiment was repeated with the same results (not shown).

### 3.6. Fermentation with ^13^C-Labeled Glycerol

**Figure 3:**
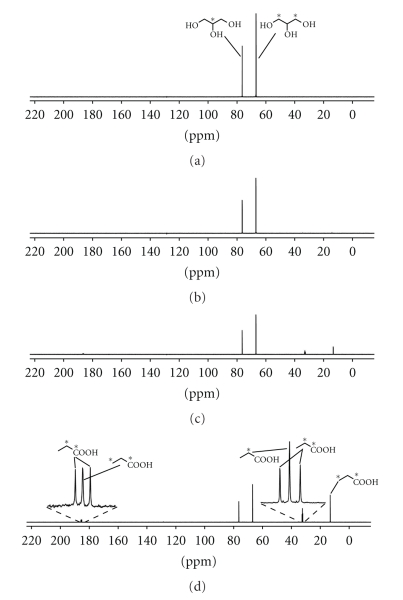
^13^C-NMR analysis of liquid cultures of *Propionibacterium jensenii* harvested after 0 h (a), 24 h (b), 48 h (c), or 96 h (d). A 1: 1 mixture of ^13^C_1, 3_-glycerol and ^13^C_2_-glycerol was used as carbon source, (∗ = ^13^C).

A fresh culture of *P. jensenii *was grown for 96 h in GLY broth prepared with ^13^C-labeled glycerol. Samples were withdrawn at 0, 24, 48, and 96 h and analyzed with respect to pH, bacterial counts, glycerol, and production of acids (Table 3). The pH of the broth at the beginning of the experiment was 6.2, and after 24 h of incubation with *P. jensenii *only a slight reduction to 6.1 was observed. After 48 h of incubation, the pH dropped to 4.9 and at the last sampling point, 96 h, it was 4.6. Initially, HPLC analysis only detected glycerol in the GLY broth, but with each following sampling point, the level of propionic acid increased at the expense of the glycerol concentration. However, the decrease in glycerol concentration was faster than the production of propionic acid, indicating the utilization of glycerol for bacterial growth supporting pathways. Each sample was also analyzed with ^13^C NMR spectroscopy to follow the glycerol conversion, and propionic acid was the only ^13^C-labeled metabolite detected (Figure 3). The concentration of propionic acid increased over time, with the highest concentration detected in the sample harvested after 96 h.  Figure 3(a) shows ^13^C-labeled glycerol, where asterisks denote the labeled carbons. The labeling pattern of the propionic acid formed is also shown by asterisks in Figure 3(d). The signals from ^13^C-labeled glycerol decreased over time, and at 48 h (Figure 3(c)), small signals from ^13^C-labeled propionic acid appeared. Figure 3(d) shows signals for propionic acid produced after 96 h of incubation. It also presents magnified regions of the ^13^C NMR spectrum and schematic pictures of the propionic acid formed with labeling at C_1_ and C_2_, C_1_, and C_3_, and a single label at C_2_. Labeling at both C_1_ and C_2_ resulted in doublet signals for both C_1_ and C_2_, caused by spin-spin coupling (^1^ *J*_C,C_ 53 Hz) of the neighboring ^13^C nuclei. Labeling at both C_1_ and C_3_ did not result in any observable signal splitting, due to the small values for ^1^ *J*_C,C_ in aliphatic carboxylic acids (often ~1 Hz) [24].

## 4. Discussion

The antifungal activity of dairy propionibacteria type strains was evaluated in the presence of glycerol. Studies were performed on both liquid and solid media and complemented with a glycerol conversion study using ^13^C-labeled glycerol. Mold inhibition in the overlay assay was observed with all bacteria, and by increasing the glycerol concentration, the inhibitory effect was enhanced. *P. freudenreichii *subsp. *freudenreichii *and *P. freudenreichii *subsp. *shermanii *showed the most inhibitory results. However, with the overlay assay, the inhibitory activity of the different bacteria against the yeast *R. mucilaginosa* was hardly affected by the increasing concentration of glycerol. In broth, molds were only inhibited by the supernatant of GLY medium cultured with *P. acidipropionici*, *P. jensenii, *and *P. thoenii. *In contrast to the solid matrix assay, the yeast under evaluation was completely inhibited by all propionibacteria when the supernatant of GLY medium was used. There is one important difference between the two experimental systems that needs to be considered. In broth cultures, the bacterial biomass is removed after 72 hours of incubation, resulting in a constant pH and metabolite concentration for the following assay, while the agar plate assay produces a continuous supply of metabolites from the growing bacteria, diffusing into both layers of agar and lowering the pH. While the continuous diffusion of propionic acid in the overlay method favored the inhibition of mold growth, the yeast was not affected to the same extent. It is known that propionic acid, as well as sodium, potassium, and calcium propionate, is an effective inhibitor of many molds but may show only weak activity against yeasts and bacteria [25].

When comparing an agar diffusion method and a broth microdilution test for determining the minimal inhibitory concentration (MIC) values of different antibiotics against *Brachyspira hyodysenteriae*, Rohde et al. [26] found a significant difference in the results obtained by both methods. The MIC values obtained were, on average, one dilution step lower for the broth dilution method. The difference observed was explained based on the divergent characteristics of the solid and liquid media, which provide different conditions for the diffusion of antibiotics or other substances [26]. The specific growth characteristics of the unicellular yeasts and the filamentous molds may also lead to different responses to antifungal conditions in diverse environments. Glycerol is a commonly used chemical to adjust water activity [1]. However, the levels of glycerol added to the agar in the plate assays gave no measurable reduction in water activity (data not shown). Indeed, the addition of 100 mM glycerol to water only decreased the water activity from 1.000 to 0.995, so a much higher glycerol concentration would be needed to cause any fungal growth inhibition.

The final pH of liquid bacterial cultures grown in the presence of glycerol was significantly lower which might have contributed to the enhanced effect observed with these cultures. The composition of the media plays an important role on the final pH of the cultures. SL medium contains sodium lactate which gives it an initial pH of 5.5–5.7. Addition of acid to this medium lowers the pH (4.7 after addition of 50 mM propionic acid) but not as much as compared to acid addition to pure water (pH 2.9 with the same acid), due to the buffering capacity of sodium lactate. Glycerol has no buffering capacity, so acids produced by propionibacteria in GLY medium decrease the pH more efficiently. 

Because propionic acid has a Pk_a_ of 4.87, at the final pH achieved in the cultured broths (pH of 4.3–4.7), more than 50% of the total propionic acid content occurs in undissociated form. The inhibitory effect of weak acids (i.e., propionic acid) is often attributed to the undissociated molecules [27]. However, the exact mode of action is still not fully understood and a number of theories exist. One suggests the prevention of growth by inhibition of the active transport into the cells [28], while another involves the negative effects of pumping protons from the acid by the plasma membrane H^+^_-_ATPase pump, blocking further growth-supporting action [29].

Previously, the sensitivity of eight spoilage fungi to three weak acids at different pH values has been reported [30]. For propionic acid at pH 5, the MIC reported for *R. mucilaginosa *was 30 mM, which clearly explains the suppressed growth of this yeast by all propionibacteria tested in liquid GLY cultures (Table 2). For molds used in this study, Lind et al. [30] reported that propionic acid had an MIC of 40 mM at pH 5.0, which is also in accordance with the results reported here for liquid cultures. *P. freudenreichii *subsp. *freudenreichii *and *P. freudenreichii *subsp. *shermanii* produced only 26 mM propionic acid, which was insufficient for total growth inhibition of the molds tested at pH around 5.0. The cultures grown in SL medium displayed a higher final pH, close to 6, which requires increased propionic acid concentrations for inhibition of fungal growth, since only approximately 7% of the propionic acid is present in its undissociated form at this pH [30].

When production of acids in the two systems used for bacterial growth (liquid and overlay assay) is considered, the two methods cannot be directly compared. Supernatants from broth cultures are homogenous and contain the total amount of the acids determined by analysis. In gel plugs, the two layers of different substrate are mixed together for acid extraction, and the extract contains the mean value from both agar substrates. In solid medium, the amount of propionic acid produced was significantly higher for three of the five strains tested when glycerol was present in the substrate. Considering that the bacteria were grown on SL agar have grown on SL for 72 hours and then provided with an additional carbon source (overlaid glycerol), the higher propionic acid yield was not surprising, also visible in Table 1. In contrast, growth in liquid SL medium resulted in more propionic acid than growth in liquid GLY medium. Strains belonging to *P. freudenreichii *species showed a more pronounced difference, producing less than half of the propionic acid amount in GLY than in SL broth. Since the buffering capacity of SL medium (sodium lactate) is absent in GLY medium, the production of propionic acid immediately lowers the pH, which can promote an inhibiting environment even for the propionibacteria itself. 

The results indicate a tendency toward a homofermentative pathway producing only propionic acid when glycerol was the energy source (Table 2). Earlier studies have shown that the use of glycerol as carbon source results in higher yield of propionic acid and less diversity in end-product composition compared to the use of glucose or lactic acid [31]. A similar observation was also made in a comparison of glycerol and glucose as carbon sources for two species of propionibacteria in batch cultures [23]. 

**Figure 4:**
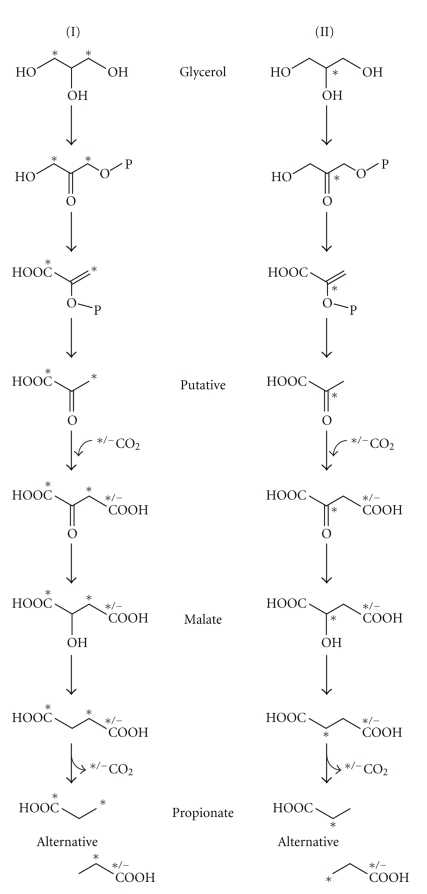
Schematic pathway for the formation of propionic acid from glycerol ^13^C labeled at C_1_ and C_3_ (I) or C_2_ (II) and ^13^CO_2_ (a by-product from ^13^C-labeled glycerol breakdown,* = ^13^C).

The observed ^13^C labeling pattern of propionic acid agrees with a previously suggested metabolic pathway for the formation of propionic acid from glycerol [31]. In this pathway, glycerol is transformed to pyruvate, via dihydroxyacetone phosphate and phosphoenolpyruvate (Figure 4). Subsequently, oxaloacetate is formed from pyruvate by the addition of CO_2_. Oxaloacetate is transformed to malate and then to succinate, and finally CO_2_ is eliminated from succinate to produce propionic acid. Propionibacteria are known to be able to use glycerol as a carbon source. Thus, during growth on ^13^C glycerol, a portion of the CO_2_ released by the bacteria is expected to be ^13^C-labeled. If ^13^C-labeled CO_2_, derived from a labeled glycerol molecule, is added to pyruvate, propionic acid ^13^C-labeled on both C_1_ and C_2_ may be formed (Figure 4), as observed in the present study (Figure 3). 

The conversion of glycerol into 3-hydroxypropionaldehyde, also known as reuterin, in LAB, involves a coenzyme B_12_-dependent glycerol/diol dehydratase. This enzyme is found in species of *Enterobacteriaceae *and *Propionibacteriaceae*, but the enzymes are immunologically distinct from each other [22]. To investigate the possibility that propionibacteria produce a reuterin-like antimicrobial compound similar to that found in LAB, we did an attempt to find equivalent enzyme gene sequences. We hybridized chromosomal DNA from strains *P. freudenreichii* subsp. *freudenreichii *and subsp. *shermanii* digested with *Bam*HI, with two adjacent ^32^P-labeled *Eco*RI-fragments derived from *L. coryniformis* Si3 according to standard methods [32]. The two fragments, together constituting approximately 5500 bp, contained the complete *pdu*A, the two *pdu*B, the *pdu*C, *pdu*D, and *pdu*E-genes. However, no signal was obtained even at low, stringency conditions (data not shown).

Combinations of antifungal propionibacteria and glycerol might be used as biopreservation systems by the food and feed industry, where fungal spoilage are of concern. In a recent study, Suhr and Nielsen [33] found that propionate was an effective mold inhibitor, except against *P. roqueforti, P. commune, *and* Eurotium rubrum*, as long as the pH and a_w_ were not too high. Both glycerol and propionic acid are approved food additives, and propionibacteria are today commonly used as starter cultures in dairy products and bread [2]. Therefore, the use of glycerol as part of a fungal inhibitory system along with propionibacteria could provide additional positive properties, since glycerol often is used as an additive to reduce the a_w_ [1]. Besides, the biodiesel industry would also benefit from new uses of the increasing surplus of glycerol [34]. Finding a qualified combination of propionibacteria and glycerol for the application as a biopreservation system in the food and feed industry is an interesting challenge for future research.

## Figures and Tables

**Table 1:** pH and acid production by 5 type strains of propionibacteria after extraction from overlay assay plates. Data in the table show mean values with one standard deviation (*n* = 4).

Bacteria	Glycerol overlay	pH	Glycerol and acid content (mM)			
			Glycerol	Lactic acid	Acetic acid	Propionic acid
*P. acidipropionici*	−*	5.5 ± 0.0	0	99 ± 6	15 ± 1	27 ± 2
	+^†^	5.2 ± 0.0	27 ± 2	102 ± 8	<10	24 ± 3
*P. freudenreichii*	−	6.0 ± 0.0	0	45 ± 6	28 ± 1	54 ± 2
*subsp. shermanii*	+	5.3 ± 0.0	12 ± 1	41 ± 4	28 ± 2	71 ± 2
*P. freudenreichii*	−	5.9 ± 0.0	0	38 ± 1	29 ± 4	50 ± 6
*subsp. freudenreichii*	+	5.2 ± 0.0	12 ± 1	36 ± 3	28 ± 3	61 ± 3
*P. thoenii*	−	6.1 ± 0.0	0	45 ± 2	29 ± 3	47 ± 3
	+	5.1 ± 0.0	12 ± 1	62 ± 6^‡^	20 ± 1	55 ± 6
*P. jensenii*	−	6.0 ± 0.0	0	34 ± 2	26 ± 3	53 ± 2
	+	5.2 ± 0.0	<10	56 ± 15	16 ± 3	62 ± 7

*The overlay agar contained no glycerol. ^†^The overlay agar contained 100 mM glycerol. ^‡^Represents only two values.

**Table 2:** Results from analysis of cultured broth after 72 h of incubation with 5 different type strains of propionibacteria. Results include bacterial counts, pH, glycerol, acids, and inhibition against three different spoilage fungi in a microtitre plate assay. Data in the table show mean values with one standard deviation (*n* = 4).

Bacteria	Broth	Bacterial count cfu/ml (log)	pH	Glycerol and acids (mM)				Supernatant inhibition*		
				Glycerol	Lactic acid	Acetic acid	Propionic acid	*Rhodotorula mucilaginosa*	*Penicillium commune*	*Penicillium roqueforti*
*P. acidipropionici*	SL	8.6 ± 0.1	5.7 ± 0.0	0	65 ± 3	22 ± 1	50 ± 2	−	−	−
	GLY	8.5 ± 0.4	4.3 ± 0.0	54 ± 1	0	0	46 ± 0	+	+	+
*P. freudenreichii subsp. shermanii*	SL	9.7 ± 0.1	6.0 ± 0.0	0	<10	39 ± 2	77 ± 1	−	−	−
	GLY	9.4 ± 0.1	4.7 ± 0.0	73 ± 1	0	0	26 ± 0	+	−	−
*P. freudenreichii *	SL	9.5 ± 0.1	5.9 ± 0.0	0	<10	39 ± 1	75 ± 0	−	−	−
*subsp. freudenrechii*	GLY	8.8 ± 0.0	4.7 ± 0.0	74 ± 1	0	0	26 ± 2	+	−	−
*P. thoenii*	SL	9.2 ± 0.1	5.9 ± 0.0	0	93 ± 4	17 ± 1	35 ± 2	−	−	−
	GLY	9.1 ± 0.1	4.5 ± 0.0	69 ± 1	0	0	33 ± 1	+	+	+/−
*P. jensenii*	SL	9.5 ± 0.2	5.8 ± 0.0	0	<10	43 ± 1	89 ± 1	−	−	−
	GLY	9.2 ± 0.1	4.3 ± 0.0	51 ± 1	0	0	49 ± 1	+	+	+

*Inhibition was interpreted a: – (no inhibition compared to the control), +/− (weaker fungal growth than control) or + (complete inhibition).

**Table 3:** Results for bacterial counts, pH, and acids from fermentation of GLY broth prepared with ^13^C-labeled glycerol by *P. jensenii*.

Sampling point	Bacterial counts cfu/ml (log)	pH	Glycerol and acids (mM)			
			Glycerol	Lactic acid	Acetic acid	Propionic acid
0 hours	7.0	6.2	105	0	0	0
24 hours	8.7	6.1	105	0	<5	<5
48 hours	9.0	4.9	74	0	0	34
96 hours	9.2	4.6	36	0	0	75

## References

[1] Adams MR, Moss MO (2000). *Food Microbiology*.

[2] Vorobjeva L (1999). *Propionibacteria*.

[3] Schnürer J, Magnusson J (2005). Antifungal lactic acid bacteria as biopreservatives. *Trends in Food Science and Technology*.

[4] Rouse S, Harnett D, Vaughan A, Sinderen DV (2007). Lactic acid bacteria with potential to eliminate fungal spoilage in foods. *Journal of Applied Microbiology*.

[5] Sathe SJ, Nawani NN, Dhakephalkar PK, Kapadnis BP (2007). Antifungal lactic acid bacteria with potential to prolong shelf-life of fresh vegetables. *Journal of Applied Microbiology*.

[6] Gerez CL, Torino MI, Rollán G, Font de Valdez G (2009). Prevention of bread mould spoilage by using lactic acid bacteria with antifungal properties. *Food Control*.

[7] Broberg A, Jacobsson K, Ström K, Schnürer J (2007). Metabolite profiles of lactic acid bacteria in grass silage. *Applied and Environmental Microbiology*.

[8] Sjögren J, Magnusson J, Broberg A, Schnürer J, Kenne L (2003). Antifungal 3-hydroxy fatty acids from *Lactobacillus plantarum* MiLAB 14. *Applied and Environmental Microbiology*.

[9] Lavermicocca P, Valerio F, Visconti A (2003). Antifungal activity of phenyllactic acid against molds isolated from bakery products. *Applied and Environmental Microbiology*.

[10] Ekinci FY, Gurel M (2008). Effect of using propionic acid bacteria as an adjunct culture in yogurt production. *Journal of Dairy Science*.

[11] Higginbotham GE, Mueller SC, Bolsen KK, DePeters EJ (1998). Effects of inoculants containing propionic acid bacteria on fermentation and aerobic stability of corn silage. *Journal of Dairy Science*.

[12] Filya I, Sucu E, Karabulut A (2006). The effects of *Propionibacterium acidipropionici* and *Lactobacillus plantarum*, applied at ensiling, on the fermentation and aerobic stability of low dry matter corn and sorghum silages. *Journal of Industrial Microbiology and Biotechnology*.

[13] Schwenninger SM, Meile L (2004). A mixed culture of *Propionibacterium jensenii* and *Lactobacillus paracasei* subsp. *paracasei* inhibits food spoilage yeasts. *Systematic and Applied Microbiology*.

[14] Ho PH, Luo JB, Adams MC (2009). Lactobacilli and dairy propionibacterium with potential as biopreservatives against food fungi and yeast contamination. *Applied Biochemistry and Microbiology*.

[15] El-Shafei K, Abd El-Gawad MAM, Dabiza N (2008). A mixed culture of *Propionibacterium thoenii* P-127, *Lactobacillus rhamnosus* and *Lactobacillus plantarum* as protective cultures in kareish cheese. *Polish Journal of Food and Nutrition Sciences*.

[16] Faye T, Brede DA, Langsrud T, Nes IF, Holo H (2002). An antimicrobial peptide is produced by extracellular processing of a protein from *Propionibacterium jensenii*. *Journal of Bacteriology*.

[17] Tawfik NF, Sharaf OM, Effat BA, Mahanna NS (2004). Preserving Domiati cheese using metabolites of *Propionibacterium thoenii* P-127. *Polish Journal of Food and Nutrition Sciences*.

[18] Gwiazdowska D, Trojanowska K (2006). Antimicrobial activity and stability of partially purified bacteriocins produced by *Propionibacterium freudenreichii* ssp. *freudenreichii* and ssp. *shermanii*. *Lait*.

[19] EBB European Biodiesel Board. http://www.ebb-eu.org/stats.php.

[20] Magnusson J (2003). *Antifungal activity of lactic acid bacteria*.

[21] Hosoi N, Morimoto K, Ozaki C (1978). Enzyme activities involved in the metabolism of 1,2-propanediol by *Propionibacterium freudenreichii*. *Journal of Fermentation Technology*.

[22] Toraya T, Kuno S, Fukui S (1980). Distribution of coenzyme B-dependent diol dehydratase and glycerol dehydratase in selected genera of *Enterobacteriaceae* and *Propionibacteriaceae*. *Journal of Bacteriology*.

[23] Himmi EH, Bories A, Boussaid A, Hassani L (2000). Propionic acid fermentation of glycerol and glucose by *Propionibacterium acidipropionici* and *Propionibacterium freudenreichii* ssp. *shermanii*. *Applied Microbiology and Biotechnology*.

[24] Kalinowski H-O, Berger S, Braun S (1988). *Carbon-13 NMR Spectroscopy*.

[25] Davidson PM, Doyle MP, Beuchat LR, Montville TJ (2001). Chemical preservatives and natural antimicrobial compounds. *Food Microbiology: Fundamentals and Frontiers*.

[26] Rohde J, Kessler M, Baums CG, Amtsberg G (2004). Comparison of methods for antimicrobial susceptibility testing and MIC values for pleuromutilin drugs for *Brachyspira hyodysenteriae* isolated in Germany. *Veterinary Microbiology*.

[27] Eklund T, Gould GW (1989). Organics acids and esters. *Mechanisms of Action of Food Preservation Procedures*.

[28] Freese E, Sheu CW, Galliers E (1973). Function of lipophilic acids as antimicrobial food additives. *Nature*.

[29] Cole MB, Keenan MHJ (1987). Effects of weak acids and external pH on the intracellular pH of *Zygosaccharomyces bailii* and its implications in weak-acid resistance. *Yeast*.

[30] Lind H, Jonsson H, Schnürer J (2005). Antifungal effect of dairy propionibacteria—contribution of organic acids. *International Journal of Food Microbiology*.

[31] Barbirato F, Chedaille D, Bories A (1997). Propionic acid fermentation from glycerol: comparison with conventional substrates. *Applied Microbiology and Biotechnology*.

[32] Sambrook J, Fritsch EF, Maniatis T (1989). *Molecular Cloning: A Laboratory Manual*.

[33] Suhr KI, Nielsen PV (2004). Effect of weak acid preservatives on growth of bakery product spoilage fungi at different water activities and pH values. *International Journal of Food Microbiology*.

[34] Behr A, Eilting J, Irawadi K, Leschinski J, Lindner F (2008). Improved utilisation of renewable resources: new important derivatives of glycerol. *Green Chemistry*.

